# Mechanocaloric Effects Characterization of Low-Crystalline Thermoplastic Polyurethanes Fiber

**DOI:** 10.3390/polym16233360

**Published:** 2024-11-29

**Authors:** Jiongjiong Zhang, Yilong Wu, You Lv, Guimei Zhu, Yuan Zhu

**Affiliations:** 1 Department of Physics, Southern University of Science and Technology, Shenzhen 518055, China; zhangjj7@sustech.edu.cn; 2 School of Microelectronics, Southern University of Science and Technology, Shenzhen 518055, China; 12232533@mail.sustech.edu.cn (Y.W.); 12031123@mail.sustech.edu.cn (Y.L.)

**Keywords:** solid-state cooling, mechanocaloric, elastocaloric, twistocaloric, thermoplastic elastomer

## Abstract

Mechanocaloric cooling/heat pumping with zero carbon emission and high efficiency shows great potential for replacing traditional refrigeration with vapor compression. Mechanocaloric prototypes that are developed using shape memory alloys (SMAs) face the problems of a large driving force and high cost. In this work, we report a low-crystalline thermoplastic polyetherurethane (TPU) elastomer fiber with a low actuation force and good mechanocaloric performance. We fabricate the TPU fiber and develop a multifunctional mechanical tester to measure both the elastocaloric and twistocaloric effects. In the experiments, the applied stress required to induce mechanocaloric effects of the TPU fiber is only 10~30 MPa, which is much lower than that of widely used NiTi elastocaloric SMAs (600~1200 MPa). The TPU fiber produces a maximum twistocaloric adiabatic temperature change of 10.2 K, which is 78.9% larger than its elastocaloric effect of 5.7 K. The wide-angle X-ray scattering (WAXS) results show that the strain-induced amorphous chain alignment and associated configurational entropy change are the main causes of the good mechanocaloric effects of the TPU fiber, rather than the strain-induced crystallization. This work demonstrates the potential of achieving low-force heat-efficient mechanocaloric cooling using thermoplastic elastomer fibers.

## 1. Introduction

Thermal management, particularly cooling for air conditioning, industrial manufacturing, and food preservation, plays a pivotal role in our daily lives. Approximately 20% of the total electricity supply around the world is consumed for space cooling and heat pumping applications. The global energy demand from air conditioning continues to rise rapidly and is projected to triple by 2050 [1]. After developments of over two centuries, conventional vapor compression technology has become the most mature and widely employed refrigeration technique, showcasing considerable cooling capacity and high energy efficiency. However, the use of hydrofluorocarbon refrigerants in vapor compression systems poses a threat to the environment [2]. At present, there exists no perfect alternative refrigerant that has features of zero greenhouse effect, high efficiency, low flammability, non-toxic properties, and other desirable traits [3].

In the realm of sustainable cooling technologies, caloric cooling using solids, characterized by zero carbon emissions and high energy-saving potential, has emerged as a promising alternative to vapor compression. The caloric cooling cycle is realized by employing materials that exhibit various caloric effects, typically arising from external physical field-driven first-order phase transitions [4,5,6]. Based on the applied fields, the developed caloric cooling methods are primarily categorized as magnetocaloric [7,8], electrocaloric [9,10], and mechanocaloric techniques [11,12,13,14]. Mechanocaloric effects are stress-induced and can be further divided into elastocaloric (by uniaxial stress) [15,16,17,18], twistocaloric (by shear stress) [19], barocaloric (by hydrostatic pressure) effects [20], and others, based on the deformation mode and stress distribution.

Mechanocaloric cooling/heat pumping has been extensively studied over the past decade [21,22,23,24,25,26,27,28] and is considered a high-priority alternative [29] to vapor compression due to its high cooling performance. Most of the mechanocaloric systems adopt superelastic shape memory alloys (SMAs) as elastocaloric components [30,31,32]. For example, NiTi SMAs remain the commonly used mechanocaloric materials due to their substantial adiabatic temperature change of over 20 K [18,33,34,35]. However, the NiTi SMAs require very large stress (normally 600 MPa~1200 MPa) to induce the phase transitions and related elastocaloric effect [33,36,37]. Such high stress levels make the mechanical actuators occupy big volumes in reported elastocaloric devices using SMAs [38,39,40,41,42], which impedes the practical applications of this novel cooling technology.

Fortunately, polymers with a low actuation force offer promise as soft and lightweight alternative mechanocaloric materials to overcome the above-mentioned challenges [13,19,43]. In contrast to the martensitic phase transformation-induced elastocaloric effect in SMAs [15], the mechanocaloric effect in polymers stems from the transition of internal molecular chains between disordered and ordered arrangements under external stress, resulting in heat release or absorption [44]. An elastocaloric effect with a temperature change of almost 10 K has been revealed in natural rubbers [13]. However, the widely used commercially available natural rubbers for elastocaloric cooling face the challenges of large deformations, low recyclability, and poor cycling stability. Thermoplastic elastomers with a large entropy change and recyclable characters have the potential to become candidates for efficient low-force mechanocaloric materials, but the comprehensive characterization of their mechanocaloric performance, including both the twistocaloric and elastocaloric effect, has not yet been fully explored.

In this work, we fabricated thermoplastic polyetherurethane (TPU) fibers and thoroughly characterized their mechanocaloric effects experimentally. We measured both the elastocaloric and twistocaloric effects of the TPU fiber by adopting uniaxial tension and torsion loading modes, respectively. With a low microstructure transition stress of only 7.5 MPa, the TPU shows an elastocaloric adiabatic temperature change of 5.7 K. In addition, the TPU fiber shows excellent twistocaloric effects with a maximum adiabatic temperature change of 10.2 K. The results in this work demonstrate the potential of using the TPU for low-force mechanocaloric cooling.

## 2. Materials and Methods

**Figure 1:**
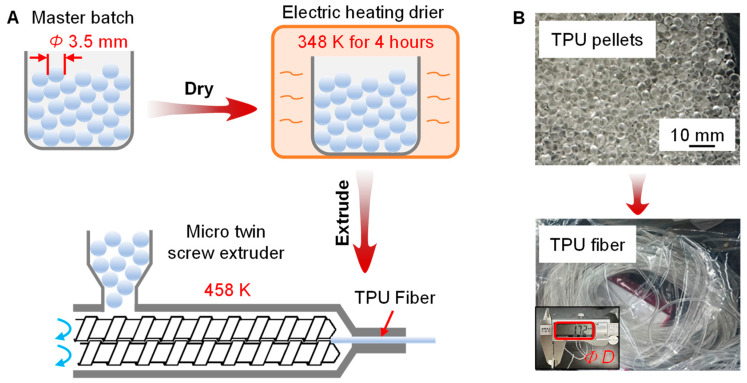
Fabrication of the TPU fiber. (**A**) Fabrication process of the TPU fiber through a micro twin-screw extruder. (**B**) Pictures of the TPU pellets and the as-spun TPU fiber with a diameter of 1.7 mm.

**Figure 2:**
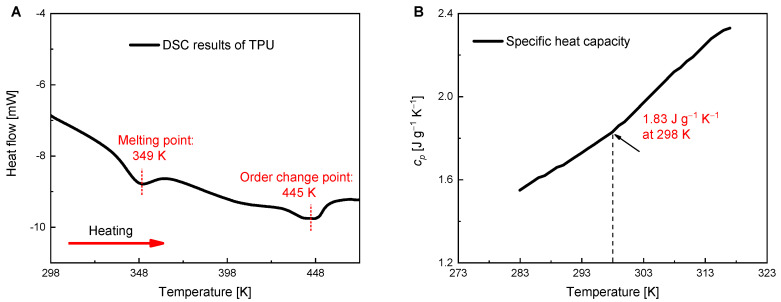
Material properties of the TPU pellets. (**A**) Heat flow of the TPU in the heating process. (**B**) Specific heat capacity of the TPU in the heating process.

To explore the potential of TPU material as an effective solid-state mechanocaloric refrigerant, we fabricate the TPU fibers using commercially available TPU pellets, as shown in Figure 1. The TPU pellets, with an average diameter of around 3.5 mm, were provided by Shanghai KunDuShiYe Co., Ltd. (Shanghai, China), and the specific model of the TPU is No. 1031. The TPU used in this study is a polyetherurethane that consists of polyether soft segments and methylene diphenyl diisocyanate (MDI)-based hard segments. More detailed material information on the raw TPU pellets is shown in Appendix A Table A1. The TPU exhibits good malleability within a certain temperature range and can be thermoformed after heating and melting. According to the differential scanning calorimetry (DSC) results (measured by TA Discovery with a heating rate of 5 K min^−1^ under a nitrogen atmosphere; the total mass of the sample is around 20 mg) shown in Figure 2, the melting temperature of the TPU pellets is about 349 K. The estimated enthalpy ∆*H*_m_ in the melting process and ∆*H*_t_ in the order change process are 5.4 J g^−1^ and 4.2 J g^−1^, respectively. Based on a theoretical maximum melting transition enthalpy of about 30 J g^−1^ for high-crystalline polyetherurethane, the content of the crystalline phase in our TPU is estimated to be about 18%. The specific heat capacity (*c*_p_) of the TPU materials is 1.83 J g^−1^ K^−1^ at a room temperature of 298 K.

In this work, the TPU fibers are prepared through melt extrusion, as shown in Figure 1A. To avoid the degrading of the TPU by water during the melting process, the TPU pellets are first dried at a temperature of 348 K for 4 h in an electric-heating air-blowing dryer (provided by Shanghai Yiheng Scientific Instruments Co., Ltd. (Shanghai, China); more details are shown in Appendix A Table A2). After drying, the TPU pellets are transferred to a micro twin-screw extruder (model of SJZS-10B, provided by Wuhan Ruiming Experimental Instrument Manufacturing Co., Ltd. (Wuhan, China); more details are shown in Appendix A Table A3) and are extruded into fibers at an elevated temperature of 458 K. The diameter of the prepared TPU fiber is 1.7 mm, as shown in Figure 1B.

**Figure 3:**
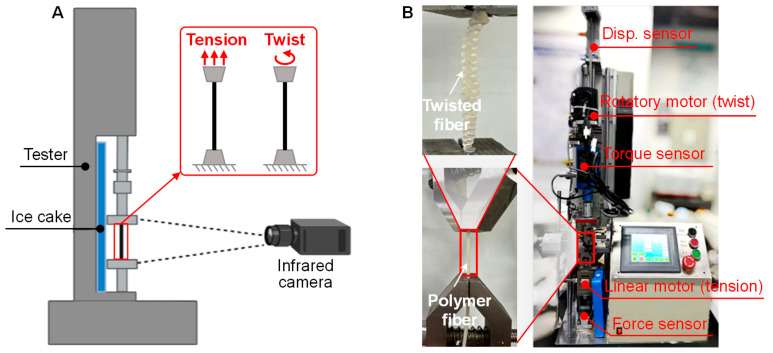
Experimental setup for mechanocaloric effect characterization. (**A**) Design of a multifunctional experimental tester for stretching and twisting the TPU fiber. (**B**) Pictures of the experimental tester and the assembly of the polymer fiber with the loading fixtures.

To experimentally characterize the mechanocaloric effect of the fabricated TPU fibers, we constructed a multifunctional loading tester, as shown in Figure 3. The experimental tester can provide two loading modes for the TPU fibers: uniaxial tension and torsion (Figure 3A). An infrared camera (Optris PI 640i with a resolution of 640 × 480; more details can be seen in Appendix A Table A4) was adopted to capture the temperature of the TPU fiber during the loading–unloading process. As shown in Figure 3B, the tester mainly consists of a rotatory motor (provided by Peking Haibohua Technology Co., Ltd., (Beijing, China) and a linear motor (provided by Shanghai Ruking Technology Co., Ltd. (Shanghai, China)). More details of the specifications of the two motors are shown in Appendix A Table A5. The force sensor, torque sensor, and displacement sensor are integrated inside the tester to record the corresponding signals. A control panel is used to set the loading parameters. Two fixtures are used to clamp the fibers in the loading process. The elastocaloric and twistocaloric effects of the TPU fiber are characterized by stretching and twisting the fiber by the developed loading tester, respectively.

## 3. Performance of Mechanocaloric Cooling

### 3.1. Elastocaloric Effect

The elastocaloric effect of the TPU fiber fabricated is characterized using our own developed mechanical tester. In the experiment, the uniaxial tensile stress is applied and removed alternately on the TPU fiber to change its molecular orientation and induce temperature variations. In the measurements, the initial length of the TPU fiber used in the experiments is 20 mm. The adopted loading speed of stretching of the TPU fiber is 300 mm s^−1^ with a corresponding strain rate of about 15 s^−1^. The maximum loading strain is 400% in the elastocaloric effect measurements. The mechanical behavior and the corresponding adiabatic temperature change of the TPU fiber were measured in this work to evaluate its elastocaloric performance.

**Figure 4:**
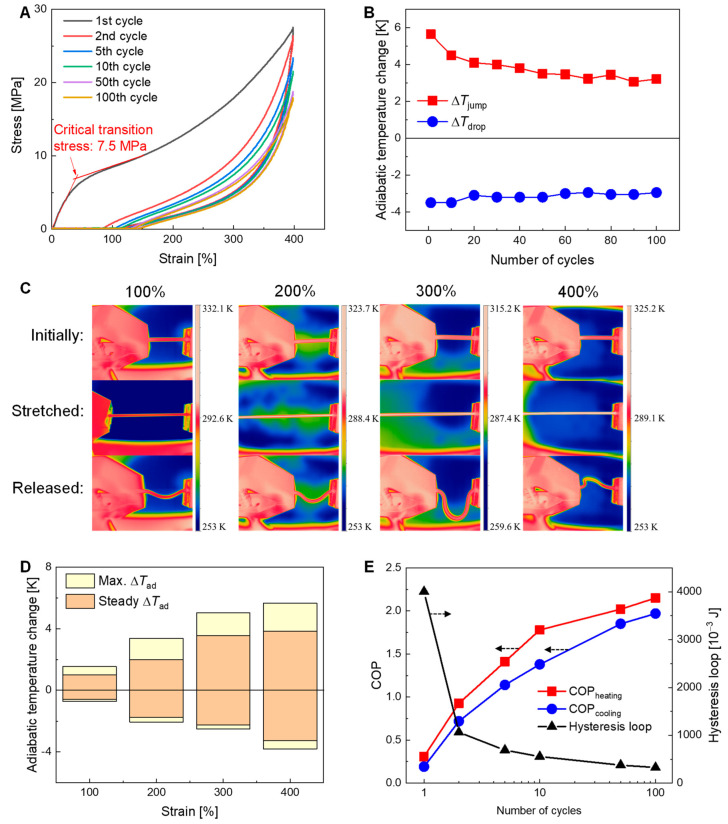
Elastocaloric effect of the TPU fiber. (**A**) Stress–strain responses of the TPU fiber under uniaxial tensile loading with a maximum strain of 400% for different numbers of loading cycles. (**B**) Elastocaloric adiabatic temperature changes of the TPU fiber at varied applied strains. (**C**) Thermal images of the TPU fiber in the loading and unloading process. (**D**) Elastocaloric temperature changes of the TPU fiber at varied applied strains. (**E**) Variation of the material COP and hysteresis loop area (net input work) of the TPU fiber with the number of loading cycles.

Figure 4A shows the stress–strain responses of the TPU fiber during the loading–unloading process with a maximum strain of 400%. It is seen that the critical stress for the microstructure transition of the TPU fiber in the first loading cycle is 7.5 MPa (Figure 4A), which is two orders of magnitude lower than that of the traditionally used NiTi SMA (~600 MPa) elastocaloric materials. With continuous cyclical loading to the 100th cycle, the critical stress and hysteresis loop area of the TPU fiber gradually decrease until a stabilized state is reached, as shown in Figure 4A.

Figure 4B shows the variation in the adiabatic temperature changes of the TPU fiber with the number of loading cycles. Thermal images of the TPU fibers at loaded and unloaded states are shown in Figure 4C. The adiabatic temperature change of the TPU fiber was measured under a special Brayton loading–unloading cycle [27]. In this cycle, the adiabatic temperature jump (Δ*T*_jump_) and drop (Δ*T*_drop_) can be obtained in the fast-loading and the fast-unloading process, respectively. In the measurements, each of the temperature data of the TPU fiber was measured ten times to ensure repeatability. The detailed errors in the data are shown in Appendix A  Table A6.

It can be seen in Figure 4B that the TPU fiber produces a ∆*T*_jump_ and ∆*T*_drop_ of 5.7 K and −3.5 K in the first loading–unloading cycle, respectively. The discrepancy between the heating and cooling abilities observed in Figure 4 can be attributed to the irreversible deformation in the loading and unloading processes. During the loading process, the polymer chains undergo a more significant degree of alignment, leading to a larger temperature change (heating). In contrast, the unloading process involves a partial relaxation of the polymer chains, resulting in a smaller temperature change (cooling). With an increased number of loading cycles, the absolute values of the ∆*T*_jump_ and ∆*T*_drop_ decrease until a stabilized state is reached. The reduction in the temperature change of the TPU fiber is due to the accumulated irreversible deformation in the loading–unloading process [45]. As shown in Figure 4D, the adiabatic temperature changes of the TPU fiber at varied maximum loading strains are measured and compared. It can be seen that the absolute value of the maximum adiabatic temperature change of the TPU fiber increases with an increase in the loading strain from 100% to 400%. In addition, the temperature change increases more rapidly with the strain from 100% to 300% than it increases with the strain from 300% to 400%.

Figure 4E shows the variation in the material coefficient of performance (COP) and the hysteresis loop area (net input work) with the number of loading cycles for the TPU fiber. The COP is defined as the ratio of the heat released/absorbed (Δ*Q*) to the input work (*W*) consumed in the loading–unloading cycle, as shown in the following Equation (1).

(1) $$COP=\frac{\Delta Q}{W}=\frac{{\rho_{m}c}_{p}\left|\Delta T\right|}{\oint \sigma d\varepsilon }$$

where *ρ*_m_ (1.12 g cm^−3^) is the mass density, *c_p_* (1.83 J g^−1^ K^−1^) is the specific heat capacity under constant pressure, *σ* is the stress, and *ε* is the strain. The hysteresis loop area enclosed by the stress–strain curve is used as the input mechanical work, as reported in the literature [46,47].

Figure 4E shows that the COP_heating_ (in the heating stage) is larger than the COP_cooling_ (in the cooling stage), which is due to the irreversibility caused by the hysteresis work. Although the absolute values of the adiabatic temperature change of the TPU fiber decrease with increased loading cycles (Figure 4B), the COP_heating_ and COP_cooling_ values increase (Figure 4E). This is attributed to the fact that the hysteresis loop area decreases more rapidly (Figure 4E) than the decrease in the internal energy change of the TPU fiber.

### 3.2. Twistocaloric Effect

The twistocaloric effect, initially demonstrated in natural rubber [19], is induced by torsional shear stress and belongs to the family of mechanocaloric effects. For polymers, torsional loading enables extensive material deformation, facilitating a more complete release of internal heat compared with the uniaxial tension loading for the elastocaloric effect.

**Figure 5:**
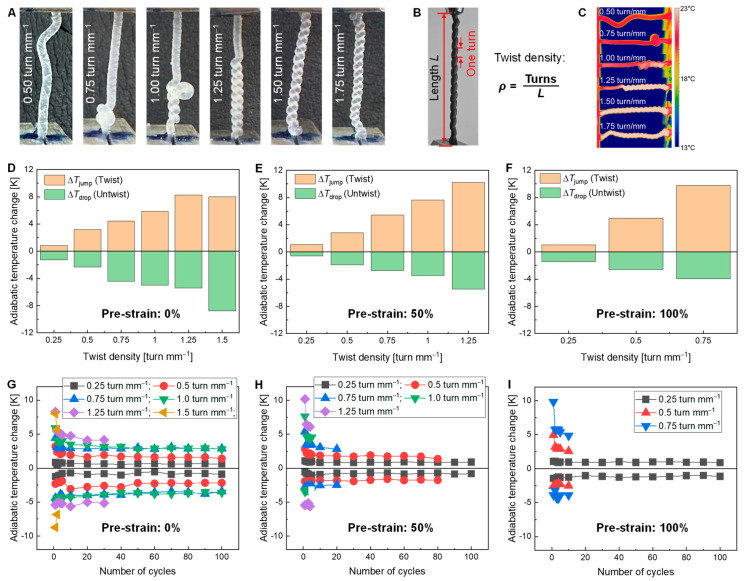
Twistocaloric effect of the TPU fiber. (**A**) Pictures of the TPU fiber during the twisting. (**B**) Definition of the twist density. (**C**) IR camera images of the TPU fiber at varied twist densities. (**D**–**F**) Twistocaloric adiabatic temperature change of the TPU fiber versus the twist density at a pre-strain of 0%, 50%, and 100%, respectively. (**G**–**I**) Variation in the twistocaloric adiabatic temperature change of the TPU fiber with the number of loading cycles at a pre-strain of 0%, 50%, and 100%, respectively.

The twistocaloric temperature change of the TPU fiber is characterized by twisting the fiber at a loading rate of 50 turns s^−1^. Before twisting, a uniaxial tensile pre-strain is imposed on the TPU fiber to make it straight and tight. The impact of the pre-strain (from 0% to 100%) on the twistocaloric performance of the TPU fiber is analyzed as shown in Figure 5.

Figure 5A shows the pictures of the TPU fiber at varied twist densities *ρ*_twist_, which reflects the extent of deformation of the twistocaloric materials. As shown in Figure 5B, the *ρ*_twist_ is defined as the following Equation (2).

(2) $$\rho =\frac{N_{turn}}{L}$$

where *N*_turn_ is the number of twisted turns (the definition of one unit turn is shown in Figure 5B) on the TPU fiber, and *L* is the length of the fiber. The temperature of the TPU fiber in the twist–untwist process is recorded by an infrared camera to characterize its twistocaloric performance, as shown in Figure 5C.

Figure 5D–F show the maximum adiabatic temperature changes of the TPU fiber under different pre-strains measured at the first loading–unloading cycle. Generally, Figure 5D–F show that the adiabatic temperature change in the TPU fiber increases with an increase in the twist density *ρ*_twist_. At a pre-strain of 0% (Figure 5D), the TPU fiber can be fully twisted to the maximum *ρ*_twist_ of 1.5 turn mm^−1^. The TPU fiber produces a maximum ∆*T*_jump_ of 8.30 K in twisting and a ∆*T*_drop_ of −8.74 K in untwisting, respectively. At the pre-strain of 50% (Figure 5E), the maximum twist density *ρ*_twist_ of the TPU fiber drops to 1.25 turns mm^−1^ and the fiber cannot be fully twisted due to the breaking at a larger *ρ*_twist_. The TPU fiber produces a maximum ∆*T*_jump_ of 10.2 K in twisting, which is increased by 78.9% compared to its elastocaloric performance of 5.7 K. Such a high twistocaloric ∆*T*_jump_ of 10.2 K is 27.5% larger than the reported elastocaloric temperature change of 8 K of TPU materials [44]. When the pre-strain further increases to 100%, the maximum twist density *ρ*_twist_ achievable by the TPU fiber decreases dramatically to 0.75 turns mm^−1^ because the fiber with a high pre-strain will be stiff and fragile in twisting.

**Figure A1:**
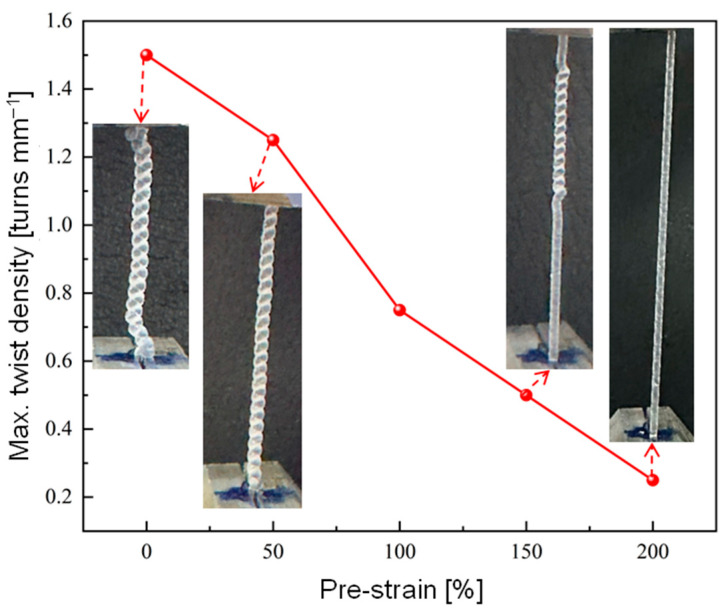
Variation in the maximum twist density allowed for the fabricated TPU fiber with an increase in the pre-strain.

Figure 5G–I show the evolution of the twistocaloric effect of the TPU fiber with an increased number of twist–untwist cycles at different pre-strains. It is observed that the adiabatic temperature change of the TPU fiber always obtains its highest value in the first twisting cycle. As the number of twist cycles increases, the adiabatic temperature change gradually degrades and eventually stabilizes. This is attributed to the accumulated irreversible break of the entanglement of chains in the TPU fiber during the loading process [48,49]. Figure 5G–I also show that the twistocaloric adiabatic temperature change of the TPU fiber consistently increases with an increase in the twist density *ρ*_twist_. However, the fatigue life of the TPU fiber decreases with an increase in the twist density *ρ*_twist_. Additionally, as the pre-strain increases, the critical twist density for the TPU fiber breaking before 100 cycles decreases (Appendix A Figure A1). Therefore, the balance between the fatigue life and the twistocaloric performance should be considered when determining the amount of applied pre-strain and twist density on the TPU fiber for practical cooling/heat-pumping applications.

### 3.3. Microstructure Analysis

**Figure 6:**
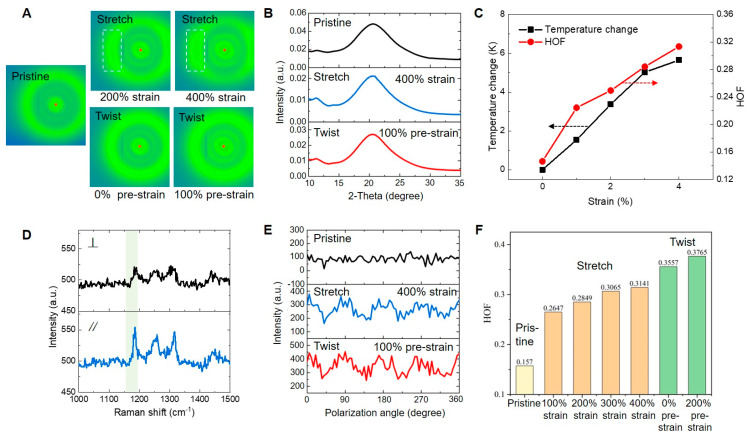
Microstructure characterization of the TPU fiber. (**A**) WAXS patterns of pristine, stretched, and twisted fibers under various strains/pre-strains. (**B**) WAXS spectra of the pristine, stretched, and twisted fibers. (**C**) Variation in the adiabatic temperature change and HOF of the fiber with the strain. (**D**) Polarized Raman spectra of the twisted TPU fiber with pre-strain of 100%. Top line: polarization perpendicular to the fiber direction. Bottom line: polarization parallel to the fiber direction. (**E**) Intensity of the 1187 cm^−1^ peak as a function of the polarization angle for pristine, stretched, and twisted fibers from the polarized Raman spectra. (**F**) HOF of pristine, stretched, and twisted fibers under various strains/pre-strains.

Wide-angle X-ray scattering (WAXS) is utilized to explore modifications in the crystallinity and alignment of polymer chains induced by stretching and twisting deformations. The WAXS is conducted by using the Rigaku Smartlab X-ray diffractometer. The experimental conditions for WAXS measurements and IR temperature measurements are carefully controlled to be consistent. Figure 6A depicts the two-dimensional WAXS patterns of pristine, stretched, and twisted fibers under various strains/pre-strains. For stretched fibers, the relative intensity of scattering rings along the stretching direction diminishes, while the relative intensity of scattering rings perpendicular to the stretching direction continues to concentrate with increasing strain. The concentrated bright spots indicate a progressive increase in fiber orientation along the axial direction [50]. Processing the two-dimensional (2D) WAXS patterns yields one-dimensional (1D) relationships between the scattering intensity *I* and the scattering angle 2*θ*. As shown in Figure 6B, an amorphous halo is detected in pristine, stretched, and twisted fibers, with its peak centered around 2*θ* = 20° and exhibiting a shoulder at 2*θ* = 12°. Unlike previous studies [44], our TPU does not exhibit significant crystallization after stretching and twisting deformations. The low crystallinity of the TPU combined with the nature of the amorphous segments results in a predominant alignment of polymer chains rather than crystallization under strain [50]. Furthermore, Herman’s Orientation Factor (HOF) [51] is used to quantify the degree of orientation of the polymers and is obtained from the azimuthal distribution of intensity, as in the following Equation (3).

(3) $$f=\frac{3}{2}\frac{\int_{0}^{\frac{\pi }{2}}I\left(\phi \right)\cos^{2}\phi \sin\phi \;d\phi }{\int_{0}^{\frac{\pi }{2}}I\left(\phi \right)\sin\phi \;d\phi }-\frac{1}{2}$$

As depicted in Figure 6C, in the case of the stretched fiber, both the temperature change and the calculated HOF increase with increasing strain. This trend indicates that the alignment of the polymer chains induces an entropy change in the TPU fiber, leading to temperature variation during the stretching process.

However, in the case of twisted fibers, no significant changes are observed in the 2D WAXS patterns, as shown in Figure 6A. The helical twisting of the fibers induces shear stress, resulting in a mixed scattering spectrum due to different chain orientations. Transmission-based measurements like WAXS lead to difficulties in characterizing the fiber’s orientation in different directions, as the signals from different orientations cancel each other out. To accurately detect local orientation in twisted fibers, a scattering technique based on reflectance is required as it does not average the scattering signal along the depth direction of the fiber. Therefore, in the subsequent sections, Raman spectroscopy is employed for a more in-depth investigation of the orientational effects of the fibers. The Raman spectroscopy was measured by using a Raman microscope with a 532 nm laser excitation. The baseline correction and peak deconvolution were performed using WiRE 4.0 software. The polarized Raman spectroscopy of the twisted TPU fibers with a pre-strain of 100% is illustrated in Figure 6D. A distinct Raman band, at approximately 1187 cm^−1^, corresponds to C-C symmetric stretching (orientation-dependent), which correlates with the local chain alignment [52]. The preferred chain orientation is perpendicular to the polarizer when this intensity ratio is minimized (top line) and parallel when it is maximized (bottom line).

Figure 6E shows the intensity of this peak as a function of the polarization angle for pristine, stretched, and twisted fibers. In the pristine fiber, the intensity of the 1187 cm^−1^ peak does not show significant changes with variations in the polarization angle. However, for stretched and twisted fibers, the intensity exhibits clear periodic variations with the polarization angle, indicating differences in their degrees of alignment. Similar to the analysis of the WAXS data, the degree of alignment can be quantitatively expressed in terms of HOF. As depicted in Figure 6F, for the stretched fiber, the HOF calculated from the polarized Raman spectra exhibits a trend similar to that calculated from the WAXS data. The twisted fiber displays a higher HOF compared to all the stretched fibers. Through polarized Raman spectroscopy, a stronger chain alignment along the local strain direction is observed in twisted fibers, which consequently explains the enhanced temperature change in the twistocaloric effect. We conclude that strain-induced amorphous chain alignment and the associated configurational entropy change are the primary causes of the observed twistocaloric effects in mechanically deformed TPU fibers, rather than strain-induced crystallization or crystalline phase changes [44].

## 4. Summary and Conclusions

In this work, we fabricate a low-crystalline TPU fiber with a low actuation force and systematically evaluate its performance of mechanocaloric cooling. The TPU fiber demonstrates a low critical transition stress of only 7.5 MPa. We establish a multifunctional mechanical tester to characterize the elastocaloric and twistocaloric effects of the TPU fiber that is fabricated. The TPU fiber shows an adiabatic elastocaloric temperature change of 5.7 K at a maximum tensile strain of 400% under uniaxial tension. With a pre-strain of 50%, the TPU fiber demonstrates a maximum adiabatic temperature change of 10.2 K at a twist density of 1.25 turn mm^−1^ in the twistocaloric effect. The microstructure analysis shows that the TPU fiber does not exhibit significant crystallization after stretching and twisting deformations due to its inherent low crystallinity. The mechanocaloric effects in the deformed TPU fiber are mainly attributed to the strain-induced amorphous chain alignment and the associated configurational entropy change rather than the strain-induced crystallization or crystalline phase changes.

The TPU fibers fabricated in our work can be mass-produced using a commercial micro twin-screw extruder for practical applications in the future. These fibers exhibit significant twistocaloric temperature changes at low actuation stress, making them viable for the development of cooling and heat-pumping devices. However, there are still some limitations within the TPU-based cooling devices. For instance, the fatigue life of TPU fibers is currently lower compared to widely used NiTi elastocaloric materials, potentially restricting the long-term operation of the device. Therefore, further efforts are needed to advance high-performance, low-stress, and long-life polymer mechanocaloric materials.

## Data Availability

The original contributions presented in this study are included in the article. Further inquiries can be directed to the corresponding authors.

## Appendix A

**Table A1:** The specifications of the raw TPU pellets provided by the Shanghai KunDuShiYe Co., Ltd. with specific model No. 1031.

Parameter	Test Method	Results
Density	ASTM D792 [53]	1.1 g cm^−3^
Hardness	ASTM D2240 [54]	80 Shore A
Tensile strength	ASTM D412 [55]	25 MPa
Viscosity	GB/T 2794 [56]	350 MPa·s
Elongation	ASTM D412 [55]	500%

**Table A2:** The specifications of the electric-heating air-blowing dryer for drying the TPU pellets.

Model	DHG9145A
Supplier	Shanghai Yiheng Scientific Instruments Co., Ltd. (Shanghai, China)
Temperature range	RT + 10~300 °C
Control accuracy	±1.0 °C
Temperature resolution	0.1 °C
Working temperature	RT + 5~40 °C

**Table A3:** The specifications of the micro twin-screw extruder for producing the TPU fibers.

Model	SJZS-10B
Supplier	Wuhan Ruiming Experimental Instrument Manufacturing Co., Ltd. (Wuhan, China)
Screw diameter	10/29 mm
Screw length	250 mm
Screw rotation speed	51 rpm
Heating power	3.5 kW
Feeding power	90 W
Nitrogen protection	Yes

**Table A4:** The specifications of the infrared camera used for capturing the thermal images.

Model	Optris PI 640i
Resolution	640 × 480
Measurement range	−20 °C–900 °C
Spectral region	7.5–13 µm
Frame Rate	Up to 125 Hz

**Table A5:** The specifications of the rotatory and linear motors of the developed multifunctional loading tester.

Model Name	Linear Motor	Rotatory Motor
Model type	HP5M60-40D30J1	HCNJ-103
Supplier	Shanghai Ruking Technology Co., Ltd. (Shanghai, China)	Peking Haibohua Technology Co., Ltd. (Beijing, China)
Maximum load rate	500 mm s^−1^	80 turns s^−1^
Maximum actuator stroke	250 mm	200 turns
Maximum load capacity	80 N	0.1 Nm
Power supply	0.4 kW	0.15 W
Load rate accuracy	0.1 mm s^−1^	0.1 turn s^−1^
Actuator stroke accuracy	1 mm	0.5%

**Table A6:** Detailed data and error of the elastocaloric temperature change of the TPU fiber.

Number of Cycles	∆*T*_jump_ [K]	Error ± [K]	∆*T*_drop_ [K]	Error ± [K]
1	5.65	0.615	−3.5	0.52
10	4.5	0.55	−3.5	0.545
20	4.1	0.615	−3.1	0.615
30	4	0.645	−3.2	0.61
40	3.8	0.555	−3.2	0.625
50	3.5	0.62	−3.2	0.64
60	3.47	1.12	−3	1.135
70	3.23	1.175	−2.95	1.145
